# Ecomorphological disparity in an adaptive radiation: opercular bone shape and stable isotopes in Antarctic icefishes

**DOI:** 10.1002/ece3.708

**Published:** 2013-08-06

**Authors:** Laura A B Wilson, Marco Colombo, Reinhold Hanel, Walter Salzburger, Marcelo R Sánchez-Villagra

**Affiliations:** 1 Paläontologisches Institute und MuseumKarl-Schmid Strasse 4, CH 8006, Zürich, Switzerland; 2 School of Biological, Earth and Environmental Sciences, University of New South WalesHigh Street, Kensington, NSW, 2052, Australia; 3 Zoological Institute, University of BaselVesalgasse 1, CH 4051, Basel, Switzerland; 4 Institute of Fisheries Ecology, Johann Heinrich von Thünen-Institute, Federal Research Institute for Rural Areas, Forestry and FisheriesPalmaille 9, 22767, Hamburg, Germany

**Keywords:** Craniofacial bone, ecology, geometric morphometrics, phylogeny, stable isotopes

## Abstract

To assess how ecological and morphological disparity is interrelated in the adaptive radiation of Antarctic notothenioid fish we used patterns of opercle bone evolution as a model to quantify shape disparity, phylogenetic patterns of shape evolution, and ecological correlates in the form of stable isotope values. Using a sample of 25 species including representatives from four major notothenioid clades, we show that opercle shape disparity is higher in the modern fauna than would be expected under the neutral evolution Brownian motion model. Phylogenetic comparative methods indicate that opercle shape data best fit a model of directional selection (Ornstein–Uhlenbeck) and are least supported by the “early burst” model of adaptive radiation. The main evolutionary axis of opercle shape change reflects movement from a broad and more symmetrically tapered opercle to one that narrows along the distal margin, but with only slight shape change on the proximal margin. We find a trend in opercle shape change along the benthic–pelagic axis, underlining the importance of this axis for diversification in the notothenioid radiation. A major impetus for the study of adaptive radiations is to uncover generalized patterns among different groups, and the evolutionary patterns in opercle shape among notothenioids are similar to those found among other adaptive radiations (three-spined sticklebacks) promoting the utility of this approach for assessing ecomorphological interactions on a broad scale.

## Introduction

Morphological disparity, a measure of the variability in morphological form, is well recognized to be unequally distributed across vertebrate phylogeny (e.g., Erwin 2007; Pigliucci 2008; Sidlauskas 2008). Evolutionary constraints place viability limits on morphological form, leaving gaps in phenotypic space; for instance, developmental programs begin at selected start points, making the achievement of some forms not possible along a particular ontogenetic pathway (e.g., Arthur 2004; Salazar-Ciudad 2006; Raff 2007; Klingenberg 2010), and the interactions between genetic or phenotypic traits can channel variation in fixed directions (e.g., Marroig and Cheverud 2005, 2010; Brakefield 2006). Understanding why phenotypic spaces possess these properties, and the evolutionary processes underlying their patterning, has long captured the attention of evolutionary biologists (e.g., Wright 1932; Simpson 1953; Gould 1989; Carroll 2005). In this regard, the study of adaptive radiations, groups that have rapidly diversified from a common ancestor to occupy a wide variety of ecological niches, has been of particular interest because these bursts of speciation have been causally implicated in generating significant portions of biodiversity, or, in other words, filling phenotypic space (e.g., Schluter, 2000; Seehausen 2007).

Classical model examples of adaptive radiation include the *Anolis* lizards of the Caribbean (e.g., Losos 2009), cichlid fishes of East Africa's great lakes (e.g., Kocher 2004; Seehausen 2006; Salzburger 2009; Santos and Salzburger 2012), and Darwin's finches from the Galápagos (e.g., Grant and Grant 2006). These systems have been well studied, and thanks to a host of empirical and theoretical approaches, some commonalities about the process of adaptive radiation have been found. All modern definitions of adaptive radiation feature a multiplication of species and adaptive diversification (Schluter, 2000; Gavrilets and Losos 2009; Glor 2010; Harmon et al. 2010). At the same time, however, the myriad and often lineage-specific interactions that guide evolutionary processes make difficult our understanding of how well these generalities may fit other, less intensively studied adaptive radiations, and much disagreement persists regarding the meaning of adaptive radiation (Harder 2001; Olson and Arroyo-Santos 2009). A main feature of adaptive radiation models is the idea that rapid diversification is possible under conditions of ecological opportunity (Schluter, 2000), and mathematical models predict that speciation rates and major ecological differences are highest at early stages of radiation (“early burst”), but decline as more and more niches become filled over time and ecological opportunity reduces (Gavrilets and Losos 2009). No two environments are the same, and the extent to which ecological conditions may place different demands on the generation and structuring of variation, and therefore impact our understanding of adaptive radiation models, is not well known (Day et al. 2013). To fill these gaps, both a wider sampling of the tempo and mode of adaptive radiations and a focus on probing the diverse boundaries of environments in which radiation has occurred are necessary.

In this study we focus on the Antarctic notothenioids, a suborder of marine perciform fishes that represent an example of adaptive radiation in an extreme environmental setting (Eastman and McCune 2000; Matschiner et al. 2011; Rutschmann et al. 2011; Lau et al. 2012). Antarctic notothenioids are endemic to the Southern Ocean, the world's coldest and iciest marine waters (Dayton et al. 1969; Hunt et al. 2003; Cheng et al. 2006). Together with the purely Antarctic Nototheniidae, Harpagiferidae, Bathydraconidae, Artedidraconidae, and Channichthyidae, the clade also includes the three ancestral families Bovichtidae, Pseudaphritidae, and Eleginopidae, represented by 11 mainly non-Antarctic species. The main radiation of the Antarctic group arose around 23 million years ago, near the Oligocene–Miocene boundary (Matschiner et al. 2011), coincident with the development of Antarctic sea ice and the progressive isolation of the Antarctic shelf. In response to changes in water temperature, Antarctic notothenioids developed adaptive features such as antifreeze glycoproteins (AFGPs) and, in one family, loss of hemoglobin that enabled them to survive and diversify in freezing waters not habitable by other teleosts (Eastman 1993; Chen et al. 1997; Hofmann et al. 2005; Near et al. 2012). Besides their taxonomic diversity, comprising 132 presently recognized species (Eakin et al. 2009), notothenioids occupy a large number of very different ecological roles (Eastman 1993). Several lineages independently evolved toward a pelagic lifestyle, a transition which, because notothenioids do not possess a swim bladder, required extensive morphological and physiological adaptations to achieve neutral buoyancy (Klingenberg and Ekau 1996; Eastman 2005). The purely Antarctic notothenioids include five major groups that differ both in their species richness and extent of morphological and ecological diversification (Eastman 2005), these are as follows: Artedidraconidae, Bathydraconidae, Channichthyidae, Harpagiferidae, and Nototheniidae. The family Nototheniidae has undergone the most ecological and morphological diversification, and includes 33 Antarctic species with life styles that range from purely benthic, epibenthic, semipelagic, and cryopelagic to fully pelagic (Klingenberg and Ekau 1996; Eastman 2005). In contrast, Harpagiferidae represents a monogeneric family of nine ecologically very similar species, and also Artedidraconidae solely comprise benthic species that mainly differ in body size (Eakin et al. 2009). Bathydraconidae are morphologically rather diverse and range from moderately robust to more elongate and delicate species, including the deepest-living notothenioids (DeWitt 1985) as well as shallow-living forms. Channichthyids are fusiform pike-like fishes, and uniquely among vertebrates they lack hemoglobin. Typically living at depths of less than 800 m, channichthyids are quite large fishes (ca. 50 cm length) and most adopt a combined pelagic–benthic lifestyle (Eastman 2005; Kock 2005).

Despite recent attention to the key features of the notothenioid radiation (e.g., Eastman 2005), very few studies have explicitly considered the evolution of morphological and environmental features among notothenioids (Ekau 1991; Klingenberg and Ekau 1996), although there exist a large number of studies of ecomorphology and functional ecology for other fishes (e.g., Lauder 1983; Bemis and Lauder 1986; Wainwright 1996; Westneat et al. 2005; Westneat 2006; Grubich et al. 2008; Mehta and Wainwright 2008; Cooper and Westneat 2009; Holzman et al. 2012). Here, we collect geometric morphometric data to describe shape evolution for a craniofacial bone, the opercle, which articulates with the preopercle and supports the gill cover in bony fish. Use of geometric morphometrics to analyze shape explicitly improves upon previous schemes of simple linear measurements (Klingenberg and Ekau 1996), which may incur complications due to size-related effects in organisms such as fishes, which are characterized by indeterminate growth. Opercle shape is indirectly related to foraging ecology because besides protecting the gill cover, the opercle plays a primary role in the suction pump phase of the respiration cycle (Hughes, 1960: Anker 1974; Lauder 1979). In a simple distinction, fish feeding on benthic prey typically use a suction-feeding mechanism, whereas those feeding on planktonic prey rely on ram feeding (Gerking 1994; Willacker et al. 2010). The ability to produce strong negative pressure gradients within the oral cavity is recognized as an important evolutionary axis of diversification (Collar and Wainwright 2006; Westneat 2006), and additional factors such as skull kinesis and jaw protrusion interact in a complex way to allow capture of aquatic prey (Holzman and Wainwright 2009). It is likely that differences in opercle size and shape along the trophic axis affect the functionality of the suction pump.

Using the opercle as an example of a functionally important and taxonomically variable craniofacial element, the aim of this study was to assess the interaction between ecology, inferred from stable isotope data, and morphology across the notothenioid clade, and to quantify the tempo and mode of ecomorphological interactions using disparity through time (DTT) and phylogenetic comparative methods. Taking advantage of its relatively well-documented development and growth (e.g., Cubbage and Mabee 1996; Kimmel et al. 2005, 2008), several studies have previously focused on the opercle, using three-spined sticklebacks as a “model” system to investigate the interplay between evolution and development. The three-spined stickleback is an example of a genealogically very recent species complex, repeatedly derived from marine ancestors after the retreat of the Pleistocene ice sheets to colonize freshwaters (Colosimo et al. 2005; Makinen and Merila 2008; Jones et al. 2012a,b). Accompanying these colonizations, opercle shape has been shown to have repeatedly evolved along the same shape trajectory in geographically distinct populations, on a relatively short time scale, following divergence from an oceanic ancestor (Kimmel et al. 2008, 2011; Arif et al. 2009). Variability in opercle shape among freshwater populations was also found to be associated with habitat, differing along the benthic–limnetic axis (Arif et al. 2009). These results demonstrate the utility of geometric morphometrics to quantify opercle shape, and imply that the globally recovered dilation–diminution trajectory of opercle shape change is most likely naturally selected. Fossils are recognized as an important component to the study of adaptive radiation (Gavrilets and Losos 2009), and the opercle model further provides an opportunity to gain insight into the temporal persistence of evolutionary patterns of shape change and their implications for the paleobiology of extinct species flocks (Wilson et al. 2013b).

## Material and Methods

### Sample and collection

All specimens photographed for this study were collected during RV Polarstern expedition ANT-XXVIII/4 to the Scotia Sea in 2012. Species identification followed Gon and Heemstra (1990) and the FAO species identification sheets for fishery purposes (Fischer and Hureau 1985). The location, date, time, water depth, and station were recorded for each trawl from which fishes were photographed (Table S1).

**Figure 1:**
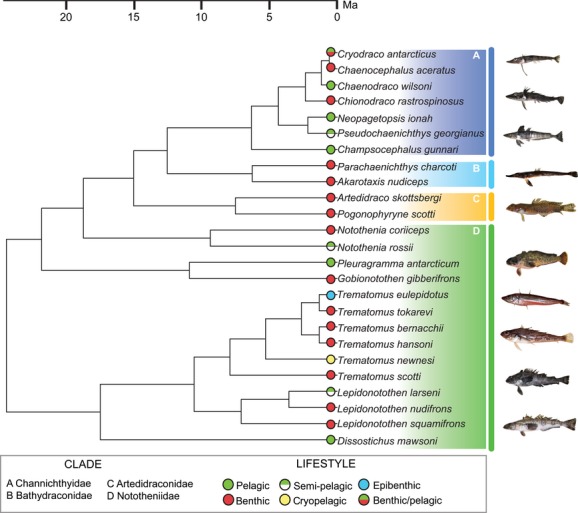
Phylogenetic relationships for the species used in this study. Filled and open circles indicate lifestyle, and major clades are highlighted and labeled. Phylogenetic relationships were based on those reported by Rutschmann et al. (2011) and Matschiner et al. (2011). Photographs of species used in this study (not to scale), from top to bottom, are as follows: *Cryodraco antarcticus*, *Chionodraco rastrospinosus*, *Champsocephalus gunnari*, *Parachaenichthys charcoti*, *Artedidraco skottsbergi*, *Notothenia coriiceps*, *Pleuragramma antarcticum*, *Trematomus eulepidotus, Lepidonotothen squamifroms*, and *Dissostichus mawsoni*. See Table 1 for further details of the study sample.

The study is based on measurements of 89 specimens from 25 notothenioid species (Table 1, Fig. 1), including representatives from each of the families Nototheniidae, Artedidraconidae, Bathydraconidae, and Channichthyidae. Each specimen was photographed in a standardized manner after being fixed in position on a flat surface using large steel needles. A Nikon D5000 camera (Nikon Corporation, Tokyo, Japan) mounted on a tripod, with the camera lens positioned such that it was parallel to the plane of the opercle, was used to capture a close-up image of the left side of the head in lateral orientation. At the initial data collection (photography) stage, each species was represented by between two and 30 individuals, as was available on the trawl, and subsequent pruning of the data set for geometric morphometric data collection was conducted to include only undamaged adult specimens, and exclude clear outliers in terms of body length to minimize intraspecific allometric variation.

**Table 1:** Specimens analyzed in this study

Group	Species	*N*	Lifestyle
Bathydraconidae	*Akarotaxis nudiceps*	1	benthic
Bathydraconidae	*Parachaenichthys charcoti*	1	benthic
Artedidraconidae	*Artedidraco skottsbergi*	1	benthic
Artedidraconidae	*Pogonophryne scotti*	1	benthic
Channichthyidae	*Chaenocephalus aceratus*	3	benthic
Channichthyidae	*Champsocephalus gunnari*	7	pelagic
Channichthyidae	*Chionodraco rastrospinosus*	7	benthic/benthopelagic
Channichthyidae	*Cryodraco antarcticus*	7	pelagic/benthic
Channichthyidae	*Neopagetopsis ionah*	1	pelagic
Channichthyidae	*Pseudochaenichthys georgianus*	3	pelagic/semipelagic
Channichthyidae	*Chaenodraco wilsoni*	4	pelagic
Nototheniidae	*Dissostichus mawsoni*	12	pelagic
Nototheniidae	*Gobionotothen gibberifrons*	10	benthic
Nototheniidae	*Lepidonotothen larseni*	1	semipelagic
Nototheniidae	*Lepidonotothen nudifrons*	2	benthic
Nototheniidae	*Lepidonotothen squamifrons*	7	benthic
Nototheniidae	*Notothenia coriiceps*	2	benthic
Nototheniidae	*Notothenia rossii*	9	semipelagic
Nototheniidae	*Pleuragramma antarcticum*	2	pelagic
Nototheniidae	*Trematomus eulepidotus*	1	epibenthic
Nototheniidae	*Trematomus hansoni*	2	benthic
Nototheniidae	*Trematomus newnesi*	2	cryopelagic
Nototheniidae	*Trematomus scotti*	1	benthic
Nototheniidae	*Trematomus tokarevi*	1	benthic
Nototheniidae	*Trematomus bernacchii*	1	benthic

### Morphometric analyses

We used an outline-based geometric morphometric approach to compare opercle shape across the notothenioid species examined. Geometric morphometrics is a useful method to analyze morphological shape, capturing data that are easily visualized in morphospace ordinations and tractable to multivariate statistical methods (e.g., Bookstein, 1991; Adams et al. 2004; Mitteroecker and Gunz, 2009). Here, and similar to a previous study (Wilson et al. 2013), an outline-based approach was chosen to assess interspecific shape variation because the curved nature of the operculum makes difficult the identification of a sufficient number of biologically meaningful, homologous, landmark points required for an accurate description of its shape across species. Eigenshape (ES) analysis is based on the definition of additional points of reference, or so-called semilandmarks (MacLeod, 1999) that are used to fill landmark-depleted regions, and in doing so enable the shape difference located in-between landmarks to be sampled, and the global aspect of a boundary outline to be evaluated (Wilson et al. 2011). ES analysis has proven to be successful in elucidating subtle shape variation in a wide variety of contexts (e.g., Polly, 2003; Krieger et al. 2007; Wilson et al. 2008; Astrop, 2011; Wilson 2013a) and is particularly suitable for this study as it affords the possibility to examine localized variation in opercular shape.

**Figure 2:**
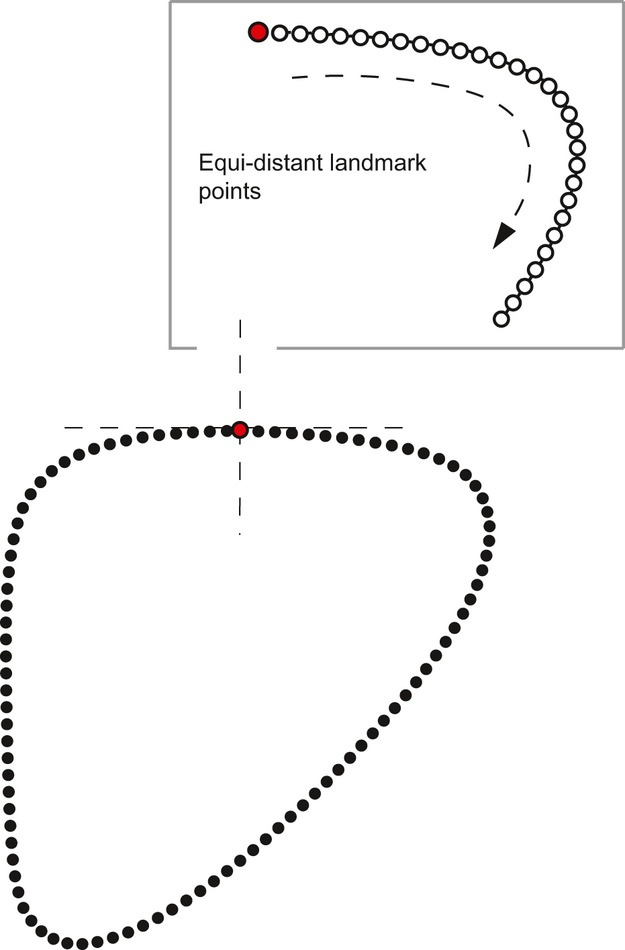
Outline-based geometric morphometric methods were used to capture the entire outline of the bone using 100 equidistant landmarks (open circles). A spatially homologous point (large color filled circle) was defined as starting point for each specimen.

For each specimen, the outline of the opercle was traced using the software tpsDig (v. 2.16, Rohlf, 2010) (Fig. 2). A type II (Bookstein, 1991) landmark was defined as the starting point for each outline, and is described as the maxima of curvature on the dorsal margin of the bone (Fig. 2). Each outline was resampled to create 100 equidistant landmark points. Cartesian *x–y* coordinates of these landmark points were converted into the phi *Φ* form of the Zahn and Roskies (1972) shape function, required for ES analysis (MacLeod, 1999). ES analysis was performed using FORTRAN routines written by Norman MacLeod (NHM London). The method is based on a singular value decomposition of pairwise covariances calculated between individual shape functions, and produces a series of mutually orthogonal latent shape vectors which represent successive smaller proportions of overall shape variation such that the greatest amount of shape variation is represented on the fewest independent shape axes. Each specimen has a series of eigenscores, representing its location along each axis, and therefore specimens can be projected into a multidimensional morphospace to visualize shape differences. Interspecific differences in shape were assessed using analysis of variance (ANOVA) coupled with post hoc tests.

### Stable isotope data

Stable isotopes of carbon and nitrogen can be used to provide insights into community trophic ecology because they show a stepwise enrichment with trophic level in marine systems (Hobson et al. 1994). The heavier isotope of nitrogen (^15^N) is enriched by 3–4 per mil per trophic level and can therefore be used to infer trophic position, whereas the heavier isotope of carbon (^13^C) is typically used to estimate the source of carbon for an organism, and practically applied to distinguish between near-shore (littoral) and open water (pelagic) environments (Post 2002). Isotope data are expressed in delta (δ) notation of per mil (‰) versus atmospheric N_2_ (AIR) and carbonate standards (V-PDB), using the equation δ = [(R_sample_/R_standard_)−1] × 1000, where R represents the ratio of the heavy to the light isotope (i.e., ^13^C/^12^C and ^15^N/^14^N) (Rutschmann et al. 2011; p4712). For all species examined, except *Akarotaxis nudiceps*, *Artedidraco skottsbergi*, *Trematomus scotti*, and *Trematomus bernacchii* for which data were not available, stable isotope data (δ^13^C and δ^15^N isotope) were compiled from Rutschmann et al. (2011) to assess the relation between opercle shape and lifestyle patterns. Rutschmann et al. (2011: File S1) sampled multiple specimens per species and we therefore computed, for each species analyzed here, an average value for δ^13^C and for δ^15^N.

The relation between shape and ecology was assessed using phylogenetic generalized least squares (PGLS) regression of δ^13^C with scores for axes ES1–ES8, and separately of δ^15^N with scores for axes ES1–ES8. PGLS uses a regression approach to account for phylogenetic relationships and assumes that residual traits are undergoing Brownian motion (BM) evolution (Rohlf 2001; Butler and King 2004; Blomberg et al. 2012). Regressions were conducted in the freely available statistical environment of R (http://r-project.org/) using the packages “geiger” and “nlme” (gls function) on a pruned data set (*N* = 21) comprising all species for which we had stable isotope values.

### Disparity analyses

To visualize the relationship between phylogeny and taxon spacing in ES space, phylomorphospaces were constructed using ES scores. For species represented by more than one specimen, average scores along each axis were used for each phylomorphospace ordination. Following Sidlauskas (2008), the plot tree 2D algorithm in the rhetenor module (Dyreson and Maddison 2003) of mesquite (Maddison and Maddison 2011) was used to construct phylomorphospaces for ES1 versus ES2 and ES1 versus ES3, comprising 75.4% of sample shape variance: subsequent axes were not plotted as each contained less than 8.6% of sample variance, and were not deemed significant under the broken-stick model (Jackson 1993). The algorithm in the Rhetenor module reconstructs the ancestral states along ES axes, plots all terminal and internal phylogenetic nodes into the morphospace, and connects adjacent nodes by drawing branches between them. Phylogenetic relationships were based on those reported by Rutschmann et al. (2011) and Matschiner et al. (2011). Branch lengths were calculated using mean value divergence dates reported by Matschiner et al. (2011).

To assess whether disparity increases rapidly at an early stage in the icefish radiation and then asymptotes, as would be predicted in a scenario of rapid early diversification (“early burst”) under conditions of ecological opportunity (Gavrilets and Losos 2009), we used DTT analyses to evaluate how shape disparity changed through time in comparison to trait evolution under a BM model. Analyses were implemented in R using the package “geiger” (Harmon et al. 2008) and the same phylogenetic framework as used for the phylomorphospace visualizations. This method calculates disparity using average pairwise Euclidean distances between species as a measure of variance in multivariate space (e.g., Zelditch et al. 2004). As input we used mean ES scores per species along axes ES1 to ES8, encapsulating 95.8% of shape variance. Following Harmon et al. (2003), relative disparities were calculated by dividing a subclade's disparity by the disparity of the entire clade. Relative subclade disparities were calculated for each node in the phylogeny, progressing up the tree from the root. At each node, the relative disparity value was calculated as the average of the relative disparities of all subclades whose ancestral lineages were present at that time (Harmon et al. 2003: 961). Relative disparity values that are close to 0.0 indicate that subclades contain only a small proportion of the total variation and therefore overlap in morphospace occupation is minimal between the different subclades, whereas, conversely, relative disparity values that are close to 1.0 indicate extensive morphological overlap. To quantify how mean disparity compared to evolution under a BM model, 1000 simulations of morphological diversification were calculated on the phylogeny, and these theoretical subclade disparity values were plotted alongside the observed disparity values for opercle shape data. A morphological disparity index (MDI) metric was obtained, representing the area contained between the line connecting observed relative subclade disparity points versus the line connecting median relative disparity points derived from BM simulations (Harmon et al. 2003). If the observed subclade disparity line plots above the BM line then the clades defined by that time slice have tended to generate higher disparity in the modern fauna than expected under the null and overlap morphospace occupied by the overall clade.

### Model fitting

BM, early burst (EB), and Ornstein–Uhlenbeck (OU) evolutionary models were fit to the data set of mean ES1 scores for opercle shape. These models describe different processes of morphological evolution on a chosen phylogeny and offer predictions about measures (e.g., disparity) of morphological trait evolution. The EB model predicts rapid morphological diversity early in the history of a group, followed by limited diversification as ecological niches are filled over time (e.g., Harmon et al. 2010). Under a BM model, trait evolution is simulated as a random walk and after each speciation event, the random walk continues independently of previous changes, and these changes are drawn from a normal distribution of zero and a variance proportional to branch length, hence phenotypic trait variance is predicted to increase with time in an unbounded fashion. The OU model is used to model stabilizing selection for a phenotypic trait value, and is similar to a BM model except traits are being pulled toward an optimal value, measured by a parameter (alpha) (Butler and King 2004; Hansen et al. 2008).

Methods for modeling evolutionary processes are largely implementable only for univariate data and therefore we chose ES1 as representative of opercle shape because it represents the maximum variance in the sample (39.9%). We repeated model fitting also for ES2 (20.6%) to assess the consistency of the best chosen model. Akaike information criterion (AIC) values were used to compare the fit of each model to the data (Akaike 1974; Wagenmakers and Farrel 2004), and specifically we report a modified version, AICc, which performs better when the number of observations per parameter is small (Burnham and Anderson 2010; Hunt and Carrano 2010). The AICc values for each model were transformed into differences from the minimum observed AICc value Δ_*i*_ (AICc) = AICc_*i*_−min AICc. The differences were then transformed into AICc weights using the calculation:


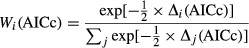


The resulting values sum to one across a set of candidate models, and can be interpreted as the proportional support received by each model (Hunt and Carrano 2010). Model fitting was conducted using the function fitContinuous() in the “geiger” package for R.

### Measurement error

Error associated with the shape variables derived from outline data sets was calculated following the methodology of Arnqvist and Martensson (1998). Landmark data collection was replicated five times each for a subset of four specimens (*A. nudiceps*, *A. skottsbergi*, *Chaenocephalus aceratus*, and *Dissostichus mawsoni*), these were selected to include representatives from each of the four families, and outlines were interpolated for the error repeats and added to the original data set. ES analysis was used to obtain shape variables and a one-way ANOVA was then performed on the outputted shape variables to detect whether the among-individual variance was greater than the within-individual (repeated) variance. The repeatability (*R*) value scales between 0 and 1. An *R* value of 0 would represent a sample in which all variance is found within individuals, whereas an *R* value of 1 would indicate all the variance is due to differences between individuals (see Wilson et al. 2011).

## Results

### Measurement error

Measurement error was calculated across the first six ES axes (ES1–ES6) accounting for 91.8% of the total sample variance, and each comprising between 3% and 39.9% of variance. One-way ANOVAs conducted on a subsampled data set including all error replicates (*N* = 20) plus original outlines resulted in *R* values of between 0.90 and 0.99, indicating a high level of replication for outline capture (Table S2).

### Patterns of opercle shape change

**Figure 3:**
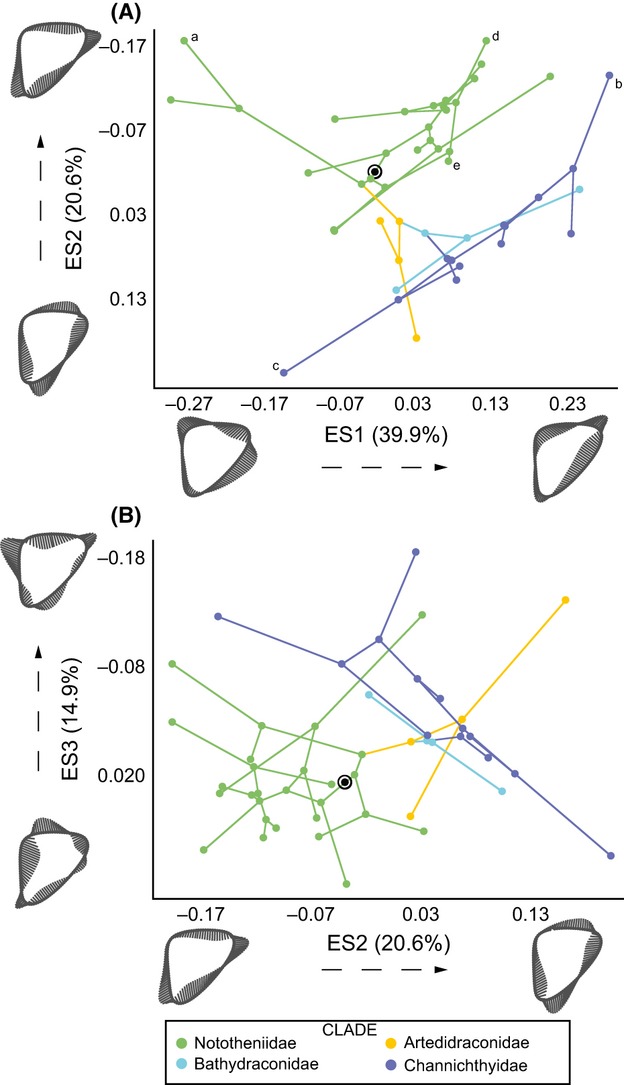
Phylomorphospace projections of notothenioid relationships on eigenshape (ES) axes ES1 and ES2 (A), and ES2 and ES3 (B) axes, describing interspecific differences in opercle shape. Branch lengths are taken from Matschiner et al. (2011), branches are colored by clade, and the root is denoted by concentric circles shaded black. Mean shape models illustrate, using vector displacements, the patterns of outline shape change associated with each axis. Tip labels, see Results for detail: a, *Notothenia rossii;* b, *Chaenocephalus aceratus;* c, *Neopagetopsis ionah;* d, *Trematomus tokarevi*; e, *Trematomus eulepidotus*.

The first three ES axes accounted for 75.3% of shape variance in the sample. Shape variance along ES1 (39.9%) was localized along two axes of the opercle outline. Negative ES1 scores reflected extension along a diagonal axis from the anterior dorsal margin to the posterior ventral margin of the bone coupled with compression along an axis from the posterior dorsal margin to the ventral tip. Conversely, positive ES1 scores reflected compression along the anterior dorsal margin and posterior ventral margin, in addition to extension along the posterior dorsal margin and ventral tip (Fig. 3A). These differences resulted in separation between species belonging to Nototheniidae, typically having negative scores along ES1, from members of Channichthyidae and Bathydraconidae, mostly characterized by positive ES1 scores (Fig. 3A). Specifically, specimens of *Notothenia rossii* (Fig. 3A, label a) had the most extreme negative scores and specimens of *C. aceratus* the greatest positive scores along the axis (Fig. 3A, label b). As for ES1, mean shape models for shape change along ES2, which represented 20.6% of shape variance in the sample, also indicated two alternating axes of extension and compression along the opercle margin. Negative ES2 scores described extension along the entire dorsal margin of the opercle and lower portion of the ventral margin, alongside compression occurring broadly along the proximal margin and the upper portion of the distal margin. Positive ES2 scores reflected changes along these axes in the opposite direction (i.e., compression instead of extension, and vice versa). Similar to ES1, *N. rossii* also occupied the most negative portion of ES2, whereas specimens of *Neopagetopsis ionah* (Fig. 3A, label c) had the greatest positive scores, equating to a lateral extension of the distal tip of the operculum, resulting in a right-angled triangle shape appearance of the bone. ES3 accounted for 14.9% of shape variance, and shape differences included a combination of variance explained by ES1 and ES2, thus resulting in two antagonistic modes of shape change occurring along each margin of the bone (Fig. 3B).

Results from ANOVA tests performed on ES1–ES8 scores, representing 95.8% of the sample variance, using “families” as groups indicated significant differences between Channichthyidae and Nototheniidae along ES1 (*F*_3,89_ = 8.525, *P* < 0.001, Bonferroni corrected), ES2 (*F*_3,89_ = 12.387, *P* < 0.001, Bonferroni corrected), and ES3 (*F*_3,89_ = 4.706, *P* < 0.001, Bonferroni corrected). Canonical variates analysis (CVA) performed on ES1–ES8 scores using all specimens in the sample, resulted in three canonical functions that explained 100% of the sample variance. Only the first canonical function (eigenvalue = 2.73) accounting for 95.6% of the variance was significant using Wilks' Lambda (*χ*^*2*^_18, 89_ = 119.46, *P* < 0.001) (Table S3).

### Disparity through time

Phylomorphospace plots of ES1 versus ES2 (Fig. 3A) and of ES2 versus ES3 (Fig. 3B) indicate a phylogenetic structuring of taxon distribution in shape space, particularly the separation of Nototheniidae and Channichthyidae and the distribution of Bathydraconidae and Artedidraconidae typically in-between those other two families. Average clade disparities for each clade were calculated from tip disparity values using the tip disparity function in the geiger package (per Harmon et al. 2003, 2008). These values were summed for each of the four clades and shape disparity was found to be highest for the Nototheniidae (0.96), followed by the Channichthyidae (0.67), the Artedidraconidae (0.16), and lastly the Bathydraconidae (0.11). Because sampling of species was unequal across the families, in part due to underlying differences in species diversity, the disparity values were subject to a simple standardization by number of taxa in each clade to yield an average per species, which was highest for Channichthyidae (0.096), followed by Artedidraconidae (0.081), Nototheniidae (0.074), and, lastly, Bathydraconiidae (0.055).

**Figure 4:**
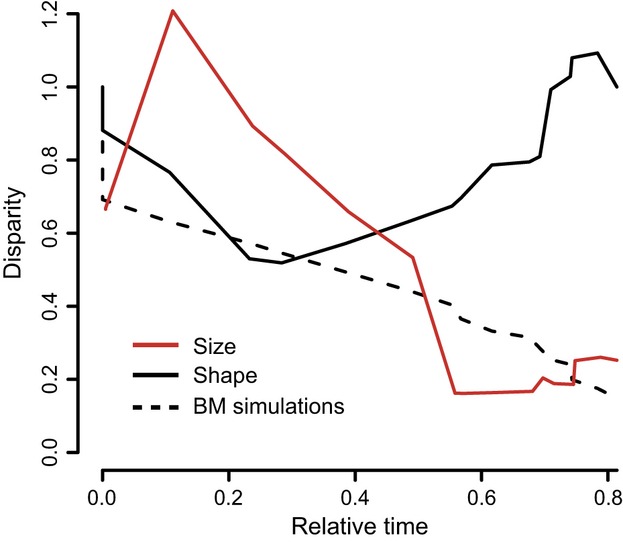
Disparity-through-time plot for opercle shape (solid black line) data, and opercle size from centroid size (solid red line) data. Mean values were used for species with more than one representative specimen. Disparity along the *Y* axis is the average subclade disparity divided by total clade disparity calculated at each internal node. The dotted line represents evolution of the data under Brownian motion (BM) simulations on the same phylogeny. Time values are relative time as per Harmon et al. (2003), whereby 0.0 represents the root and 1.0 represents the tip. The most recent 20% of the plot was omitted to avoid the effect of “tip overdispersion” due to missing terminal taxa (Muschick et al. 2012).

The DTT method was used to assess how opercle shape and size disparity compared with expected disparity based on simulations using a neutral evolution BM model (Fig. 4). Overall, shape disparity using ES scores reflecting the positioning of taxa in multivariate shape space is greater than expected by BM simulations. A similar result is obtained using only size disparity. MDI values, calculated as the area contained between the solid and dotted lines in Figure 4 or in other words the observed relative disparity points versus the line connecting median relative disparity points from the BM simulations, were similar for shape (0.341) and size data (0.453).

### Evolutionary models

The fit of the EB, OU, and BM models was assessed using the Akaike information criterion corrected for small sample size (AICc), which can be used to compare models that have different numbers of parameters (BM has two parameters, OU has three) and therefore have noncomparable log likelihoods. AICc values indicate that the best fit to ES1 shape data was the OU model (AICc = −23.02) followed by the BM model (AICc = −19.21) and lastly the EB model (AICc = −16.59) (Table 2). A similar result was found for ES2, also best supported by OU (AICc = −33.70), followed by BM (AICc = −21.69), and least supported by the EB model (AICc = −19.06). Results of AICc weight calculations indicated a comparatively high probability that the OU model (0.84) was the best model given the data and the set of candidate models (Table 2).

**Table 2:** Comparison of evolutionary models fit to opercle shape data (ES1). Akaike weight was calculated from AICc

Model	AIC	AICc	Log L	Akaike weight
Early Burst (EB)	−17.79	−16.59	11.89	0.034
Brownian Motion (BM)	−19.79	−19.21	11.90	0.125
Ornstein –Uhlenbeck (OU)	−24.23	−23.02	15.11	0.841

### Patterns of shape change in relation to habitat and trophic niche inferred from stable isotope data

**Figure 5:**
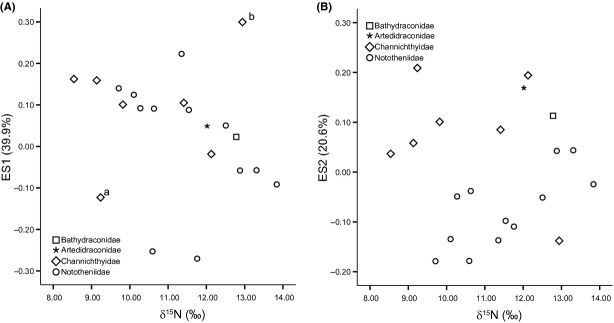
Mean shape scores for each notothenioid species along eigenshape (ES) axes ES1 (A) and E2 (B) plotted against mean δ^15^N values, denoted per mil (‰), taken from Rutschmann et al. (2011). Tip labels, see Results section for further detail: a, *Neopagetopsis ionah;* b, *Chaenocephalus aceratus*.

**Figure 6:**
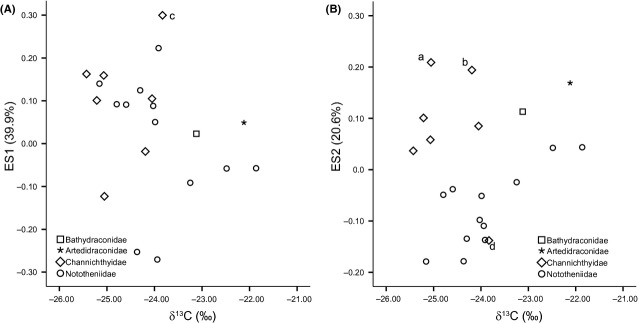
Mean shape scores for each notothenioid species along eigenshape (ES) axes ES1 (A) and E2 (B) plotted against mean δ^13^C values, denoted per mil (‰), taken from Rutschmann et al. (2011). Tip labels, see Results section for further detail: a, *Neopagetopsis ionah;* b, *Cryodraco antarcticus*, c, d, *Chaenocephalus aceratus*.

**Figure 7:**
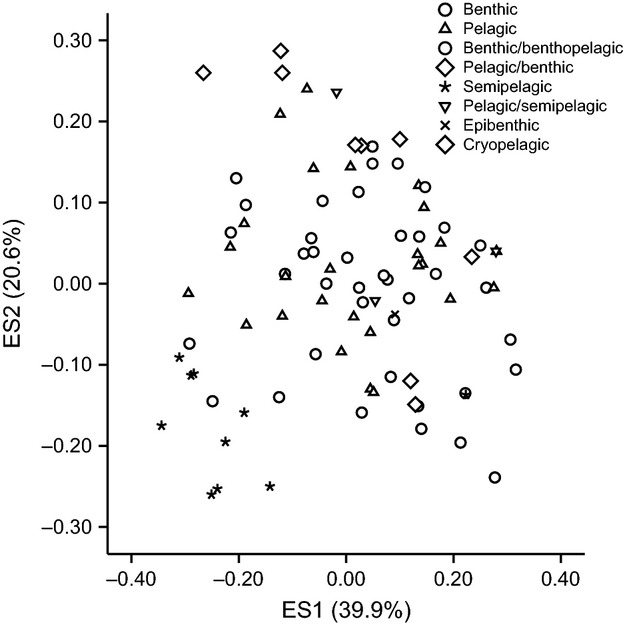
Plot of eigenshape (ES) axes ES1 and ES2 representing 60.5% of the sample variance. Markers indicate feeding strategy taken from literature sources (Gon and Heemstra 1990; Reid et al. 2007; Rutschmann et al. 2011).

A significant relationship was not found for results of PGLS regression analyses using stable isotope values for δ^13^C and δ^15^N against the matrix of mean scores along ES1–ES8 for all species (*r*^2^ < 0.15, *P* < 0.60). Members of the Channichthyidae and the Nototheniidae showed the greatest amount of spread along ES1 and along δ^15^N values (Fig. 5A) and a general, although not significant (*P =* 0.1493), trend of lower ES1 scores associated with higher δ^15^N could be observed, indicating that species inferred to occupy higher trophic levels typically had opercles with elongated posterior portions of the dorsal margin and that tapered more sharply along the entire posterior margin (see Fig. 3 top-right mean shape model), although this was not evident for ES2 scores (Fig. 5B). Rutschmann et al. (2011) previously noted that species with lower δ^13^C values were typically classified as pelagic, whereas benthic species were found to have higher δ^13^C values. Specific regions of morphospace were not exclusively occupied by benthic or pelagic species (Fig. 6). For instance, bathydraconids and artedidraconids are considered the most benthic families within Notothenioidei (La Mesa et al. 2004), but occupied broadly average scores on ES1 (Fig. 6A) and slightly higher than average scores on ES2 (Fig. 6B), although species with the highest ES2 scores occupied either a pelagic (*N. ionah*, Fig. 6B, label a) or benthopelagic niche (*Cryodraco antarcticus*, Fig. 6B, label b). Of note, *C. aceratus*, an exception among the largely pelagic Channichthyidae, is considered a benthic predator, mainly feeding on *Champsocephalus gunnari* (Reid et al. 2007), and is found to occupy separate regions of ES1 (high positive score, Fig. 6A, label c) and ES2 (high negative score, Fig. 6B, label d) reflecting a slightly different opercle morphology to other members of the group. Labeling of specimens according to their feeding strategy indicates a broad overlap in opercle morphology between benthic and pelagic species, occupying mostly the area of −0.20 to 0.20 along ES1 by −0.10 to 0.10 along ES2 (Fig. 7). Semipelagic species, represented by *Lepidonotothen larseni* and *N. rossii* have low ES1 and ES2 scores, forming a group slightly distinct from the benthic and pelagic species (Fig. 7) and equating to an opercle with an anterior margin tapering along its length in a posterior direction such that its most ventral tip is somewhat shifted posteriorly, compared to species with higher ES scores on these two axes.

## Discussion

We investigated the evolution of opercle shape in the adaptive radiation of notothenioids by quantifying shape disparity, phylogenetic patterns of shape evolution, and ecological correlates in the form of stable isotope values to assess how ecological and morphological (shape) disparity are interrelated. Our focus on the evolutionary morphology of a craniofacial bone addresses how shape disparity data may inform our growing understanding of the features that define the adaptive radiation model or patterns that may be uncovered across different groups.

Our main findings are that (1) DTT results show opercle shape and size disparity for subclades tended to generate higher disparity in the modern fauna than would be expected under the neutral evolution BM model (Fig. 5), and evolutionary model comparisons indicate that the OU model is the best fit to our data and the “early burst” model is the least well supported, (2) the main evolutionary axis of opercle shape change (ES1) reflects movement from a broad and rather more symmetrically tapered opercle to one that narrows along the distal margin, but with only a slight shape change on the proximal margin, (3) the distribution of taxa in shape space ordinations reveals a broad diversity of realizable opercle morphologies (Fig. 3) and phylomorphospace projections show clear phylogenetic groupings for opercle outline shape and a wide distribution of morphospace occupation for members of the family Nototheniidae, particularly extended by species belonging to the genus *Notothenia*, which occupy a portion of morphospace unexplored by other species (Fig. 4), and (4) a significant relationship was not detected between opercle shape and isotope values using PGLS regression.

### Opercle shape and benthic/pelagic trends

In contrast to other morphological features that have been quantified in the classical examples of adaptive radiation such as cichlids and *Anolis* lizards, the study of evolutionary patterns of craniofacial bone shape has received comparatively less attention as previous studies have first focused on traits that are the likely candidates to display ecologically or functionally related variability, such as whole-body shape (Barluenga et al. 2006; Clabaut et al. 2007; Berner et al. 2010; Harrod et al. 2010) or the jaw apparatus (Muschick et al. 2011, 2012). A notable exception are the studies of Kimmel and others that have examined opercle variability (Kimmel et al. 2008; Arif et al. 2009; Kimmel et al. 2010) in different populations of three-spined sticklebacks (but see also Willacker et al. 2010), a well-established subject of study for speciation research (e.g., Schluter and McPhail 1992; Shapiro et al. 2004; Colosimo et al. 2005). The major axis of shape variation found in the opercle of three-spined stickleback populations from Iceland to diverse locations along the western coast of North America reflects a dilution–diminution mode of shape change (Kimmel et al. 2008, 2011), that is, an anterior–posterior stretching coupled with a dorsal–ventral compression of the outline shape. This pattern explains change between freshwater and marine populations, whereas the second axis of shape change (PC2: Kimmel et al. 2011) is attributed to foraging ecology along the benthic–limnetic axis and translates to an overall widening of the opercle. Our mean shape models indicate that for notothenioids the major axis of shape variability (=ES1) in the sample reflects a similar extension and compression, but these axes of shape change are not strictly in the craniocaudal and anterior–posterior direction, instead being slightly offset (Fig. 3). The general trend along ES2 also reflects a widening and narrowing of the opercle margin, as for sticklebacks (Kimmel et al. 2011). A lack of clear phylogenetic segregation in Figure 5A also indicates that along ES1 members of the Channichthyidae and Nototheniidae therefore have evolved broadly similar opercle shapes in relation to their position along the pelagic–benthic axis (Fig. 6A). Besides sticklebacks, differences in feeding mechanism are already known to be reflected in body shape and bone morphology among benthic and limnetic morphotypes in cichlids (e.g., Barluenga et al. 2006; Clabaut et al. 2007; Muschick et al. 2012). The finding that benthic species in this study generally have an extended posterior margin of the opercle compared to pelagic species is consistent with the results of Klingenberg and Ekau (1996) who examined a series of body measurements among several Nototheniidae belonging to the subfamilies Trematominae and Pleuragramminae. Klingenberg and Ekau (1996) found that benthic species had larger values for head width, which we here may consider to be reflected in the opercle by an extension of the posterior margin, and mouth length measures than pelagic species. Those authors speculated that these morphological features may reflect the larger sized prey available for consumption in benthic environments.

### Evolutionary model fitting

Our data indicate a strong preference for the OU model, which models selection to a single (global) optimum for all species, and suggests that the here observed disparity patterns may result from an adaptive peak or constraint, as highlighted more broadly in several other fish radiations, such as cichlids (Young et al. 2009; Cooper et al. 2010) and in agreement with a recent broad-scale geometric morphometric study of cranial and postcranial bone shape in actinopterygians (Sallan and Friedman 2012). Assuming that a single global optimum morphology is indeed accurate for notothenioids and given the benthic/limnetic habitat variation in the clade (Rutschmann et al. 2011), one would not expect an association of opercle shape with habitat or diet, which is supported here by a lack of significant relationship between isotope values and opercle shape data. The OU model expects more evolution to be apparent on later branches of phylogeny as selection to the optimum would result in phylogenetic signal generated from evolution at earlier branches being erased. Although the OU model supports the presence of an optimum, this conclusion must be taken cautiously here because the DTT results indicate disparity is concentrated within subclades, that is, to say closely related species differ considerably in morphology. This conflicts with convergence to a single optimum (alpha), and hence we suggest support for the OU model may rather indicate loss of phylogenetic signal due to potentially rapid divergence rather than convergence to an optimum.

At early stages of an adaptive radiation it is predicted under the “early burst” model that measures of disparity are high, followed by a subsequent drop in those values as time passes and available niche space falls to zero (e.g., Seehausen 2006; McPeek 2008). Model comparison results indicate that our data fit least well to this “early burst” model, which had the highest AICc value of all three models tested. Also, although we do find early peaks in opercle shape and size disparity (Fig. 4), which would be indicative of the rapid, early filling of empty niches, our plot does not support an “early burst” scenario (e.g., Gavrilets and Vose 2005) because we find a second peak in disparity occurring later in relative time (before 0.8, Fig. 4), and under an “early burst” scenario there would be little opportunity for subsequent ecological diversification in subclades (Harmon et al. 2003; Burbrink and Pyron 2010).

The second peak in disparity corresponds to the subclade within the family Nototheniidae including species of *Trematomus*, and the subclade comprising all representative species of the Channichthyidae with the exception of *Champsocephalus gunnari* (Fig. 1). When examining the phylomorphospace plots for ES1 and ES2 (Fig. 3A), morphospace occupation for the Channichthyidae is considerably extended by two taxa: *N. ionah* that displays low ES1 values and high ES2 values (Fig. 3A, label c) and *C. aceratus* that displays high ES1 values and low ES2 values (top right of Fig. 3A, label b). These two species may thus be contributing considerably to high values of disparity later in the DTT plot. Along with species of Notothenia, *N. ionah* also appears as an outlier on plots of δ^15^N versus ES1 (Fig. 5A, label a), falling well below the majority of taxa in that plot. Similarly, the high score along ES1 for *C. aceratus*, which as a top benthic predator (Kock 2005; Reid et al. 2007) stands out among the other largely pelagic channichthyids, results in that species being located outside (above) the main group in Figure 5A (label b). In the case of *Trematomus*, here represented by six species, Rutschmann et al. (2011) showed that species of this genus were differentiated in isotopic signatures, indicating trophic niche separation within the genus or a large niche space, and reports of stomach contents for different species corroborate this finding (Brenner et al. 2001). Within our sample, the phylomorphospace plot indicates considerable variation particularly in ES2 scores among members of *Trematomus*, especially *T. tokarevi* (benthic, Fig. 3A label d) compared to *T. eulepidotus* (epibenthic/pelagic, Fig. 3A label e), and these differences may have contributed to elevated disparity for that node. Near et al. (2012) conducted a series of DTT analyses on buoyancy measures for 54 species of notothenioids and similarly their plots (Near et al. 2012: Fig. 3A–C) also revealed a second peak in disparity, particularly for Channichthyidae and species of *Trematomus*, which those authors related to the repeated colonization of benthic, epibenthic, semipelagic, and pelagic habitats among closely related lineages. The latter is thought to have happened as a consequence of the repeated creation of open niches following extinctions caused by icebergs and glaciers scouring the continental shelf and decimating near-shore fauna (Tripati et al. 2009; Near et al. 2012).

More broadly, the lack of an “early burst” pattern in our data set fits with the results of Harmon et al. (2010), who performed a broad survey of 49 animal clades and found little evidence of an “early burst” model of morphological change, and recently Ingram et al. (2012) suggested that this may be explained by the ubiquity of omnivory in natural food webs. Ingram et al. (2012) found that the “early burst” scenario was not detected for clades containing many omnivorous species that fed at multiple trophic levels; a feature common also for notothenioids, which include several species that feed opportunistically throughout the water column (e.g., Eastman 2005). Although omnivory was suggested as one possible determinant of the adaptive burst scenario, a general trend hinted by those results is that the persistence of an “early burst” pattern may be related to the relative extent to which niche axes (such as diet, microhabitat, and climate) are distinct and stable over time (Ingram et al. 2012).

### Patterns of diversification in notothenioids

The constituent groups of the notothenioid radiation have undergone different amounts of ecological and morphological diversification, with some, such as the artedidraconids that are all sedentary benthic fishes, displaying little (Eastman 2005). Our disparity values and phylomorphospace plots to some extent reflect these patterns, particularly for the notothenioids, which display the highest disparity values and the most expanded occupation of morphospace (Fig. 3). Notothenioids are ecologically diverse and include benthic (around 50% of within-group species diversity, Eastman 1993), epibenthic, semipelagic, cryopelagic, and pelagic forms. They are also the only group containing species that have so far been determined as neutrally buoyant (*Pleuragramma antarcticum* and *D. mawsoni* are examples in our study), a feature that has been achieved, despite not possessing a swim bladder, through reduced skeletal mineralization and lipid deposition (DeVries and Eastman 1978; Eastman and DeVries 1982; Eastman 1993). Most distinct in our morphospace plots is the location of *Notothenia* species that typically have an opercle that widens at the posterior margin (ES1) and has a posteroventrally tapering dorsal margin (see top-left mean shape model, Fig. 3A). Representing the opposite end of the body mass scale compared to the neutrally buoyant members of the Nototheniidae, species of *Notothenia* are large, heavy fishes that are able to move up and down in the water column to feed on both pelagic and benthic prey, and are able to alter their diet in relation to prey availability (e.g., Fanta et al. 2003). *Notothenia coriiceps*, for example, is known to feed on macroalgae, most likely to ingest also the associated amphipods more efficiently (Iken et al. 1997; Fanta et al. 2003), when its preferred food source of krill is unavailable. *Notothenia rossii* also ingests different food during its juvenile stages, switching from a pelagic to largely benthic habit in adulthood, which may have further implications for opercle and craniofacial bone development in general. Burchett (1983) examined this ontogenetic shift from pelagic to benthic lifestyle and found an associated change in head shape (length and diameter) and a deepening of the body over the course of ontogeny. The main result of the foraging habit versus opercle shape plot, showing broad overlap in opercle morphology among different foraging categories (Fig. 7), is perhaps not unsurprising, given the dietary plasticity of many notothenioids (Eastman 2005), the aforementioned *Notothenia* being an excellent example (e.g., Foster and Montgomery 1993). The most logical reasoning behind the range of morphotypes is that notothenioids inhabit an ecosystem with relatively low species diversity and reduced competition, both of which would not act to accelerate ecomorphological divergence (Eastman 2005) to the degree found among other radiations.

## Conclusions

A major impetus for the study of adaptive radiations is to uncover generalized patterns among different groups. In this way, common features may speak for the importance of a given process in the generation of morphological diversity (Gavrilets and Losos 2009). Here, we use outline-based geometric morphometrics to quantify opercle shape across notothenioids. We identify axes of shape change, particularly a widening of the opercle bone, that have been recovered in other adaptive radiations (three-spined sticklebacks) and a trend in opercle shape change along the benthic–pelagic axis, underlining the importance of this axis for diversification in notothenioids. We find that opercle shape and size disparity for subclades tended to generate higher disparity in the modern fauna than would be expected under neutral evolution, and that the OU model best fits the evolution of opercle shape. Support for the OU model may reflect loss of phylogenetic signal due to potentially rapid divergence. Opercle shape represents one of few features that can be quantitatively assessed for both extant and extinct species flocks (Wilson et al. 2013), and therefore provides an especially useful opportunity for integrative study between evolutionary biology and paleontology (e.g., Sánchez-Villagra 2010; Wilson 2013b), an approach that has yet to be fully explored in the context of adaptive radiation, and one that holds potential to yield valuable insights into modes of species diversification in deep time.

## Supporting Information

**Table S1.** Specimens analysed in this study.

**Table S2.** Measurement error results for ES1–ES6 calculated from one-way ANOVAS (df = 1, 39).

**Table S3.** Results of canonical variates analysis (CVA) on complete sample, using “families” as groups.

**Table S4.** Information about the trawls from which photographed specimens were collected.
